# Body Image as Well as Eating Disorder and Body Dysmorphic Disorder Symptoms in Heterosexual, Homosexual, and Bisexual Women

**DOI:** 10.3389/fpsyt.2019.00531

**Published:** 2019-08-05

**Authors:** Alina T. Henn, Christoph O. Taube, Silja Vocks, Andrea S. Hartmann

**Affiliations:** Department of Clinical Psychology and Psychotherapy, Osnabrück University, Osnabrück, Germany

**Keywords:** body image, women, sexual orientation, discrimination experiences, involvement with the lesbian community, eating disorder symptoms, body dysmorphic disorder symptoms

## Abstract

Body image disturbance is a core symptom of eating disorders (EDs) and body dysmorphic disorder (BDD). There is first evidence that females’ body image differs depending on sexual orientation, with heterosexual women (HEW) appearing to show more body image disturbance symptoms than homosexual women (HOW). Such disparities might be moderated by everyday discrimination experiences and involvement with the lesbian community. However, to date, there has been no comprehensive assessment of a broad range of body image facets such as drive for thinness, leanness, and muscularity; body avoidance; body checking and body dissatisfaction; and ED and BDD pathology as well as moderating factors. Moreover, studies have often neglected bisexual women (BIW). A total of *N* = 617 women (*n* = 180 HOW, *n* = 322 HEW, *n* = 115 BIW) completed an online survey assessing the various facets of body image, ED and BDD pathology, discrimination experiences, and involvement with the lesbian community. Significant group differences were found regarding drive for leanness and thinness, body checking, investment behavior, and body ideal (all p<.05). BIW showed significantly more body checking than HOW. Compared to HEW, HOW reported a significantly lower drive for leanness and thinness as well as compared to HEW and BIW less investment behavior. HOW preferred a body ideal with significantly more body fat than did HEW (all p<.05). In contrast, no differences emerged in body dissatisfaction, drive for muscularity, body-related avoidance, ED and BDD pathology, and body image disturbance (all p>.05). In all groups, discrimination experiences were positively related to ED and BDD pathology and to body image disturbance (all *p* < .05); however, discrimination was significantly correlated with more body image facets in HEW than in HOW or BIW. Involvement with the lesbian community was positively correlated with a larger ideal body size in HOW (*p* < .05) and negatively correlated with drive for muscularity in BIW (*p* < .05). Despite the group differences in several body image facets, we found no consistent evidence of increased vulnerability to body image disturbance or associated pathology depending on sexual orientation. However, in HEW, discrimination experience might pose a risk factor for the development of body image–related pathology and single facets of body image disturbance.

## Introduction

Body image describes the mental representation of the size, shape, and form of one’s own body as well as the feelings regarding these characteristics (1). It shows a strong positive association with self-esteem (2) and psychosocial quality of life (3). Body image disturbance is a hallmark characteristic of eating disorders (EDs) (4) and body dysmorphic disorder (BDD) (5). Moreover, it has been shown to be a risk factor for the development and maintenance of EDs (6) and BDD (5).

Body image disturbance is a multidimensional construct comprising a perceptual component, e.g., overestimation of one’s own body size and body fat (7, 8) and underestimation of one’s muscularity (9); a cognitive–affective component; and a behavioral component (4, 10) (11). The cognitive–affective component includes negative thoughts, attitudes, and feelings towards one’s own body, which can manifest as body dissatisfaction, disgust, shame, or sadness (7, 12, 13). The behavioral component describes body-related behaviors (7) such as investment in one’s own body in terms of dieting or exercise, appearance fixing (14), body-related avoidance (15, 16), and body checking (17).

In general, women have a more negative body image than men [e.g., (18, 19)], with up to 80% of females reporting dissatisfaction with their own bodies (20). Additionally, women are also more likely to show ED [e.g., (21, 22)] and BDD symptoms (23), as well as full-syndrome ED (24). Besides age, other intraindividual characteristics such as sexual orientation have an impact on body image disturbance [e.g., (4, 12)]. However, previous findings are inconsistent, or results are missing in general regarding the influence of women’s sexual orientation on the different components of body image disturbance.

Research examining the cognitive–affective component of body image disturbance has revealed a significantly lower drive for a thinner body (drive for thinness) in homosexual women (HOW) than in heterosexual women (HEW) (25–29), although some studies have reported similar levels (30, 31). To date, no study has investigated the association between sexual orientation and drive for leanness, i.e., the preference for a thin and well-toned body with as little body fat as possible (32). However, a recent study reported a higher drive for muscularity (33) in HOW and bisexual women (BIW) than in HEW (31). In terms of attitudes and emotions towards one’s own body, the majority of recent studies reported a lower degree of body dissatisfaction in HOW than in HEW (34–36), although some studies reported similar levels of dissatisfaction in both groups [e.g., (31, 37)]. Notably in this context, some studies did not report any associations between sexual orientation and body mass index (BMI) [e.g., (38, 39)], while others found a higher BMI in HOW compared to HEW [e.g., (34, 40, 41)], which might account for the aforementioned findings (41). Additionally, studies employing such rating scales revealed that HOW prefer a body ideal with significantly more body fat compared to HEW (34, 42–44), although again, other studies found evidence of a similar body ideal among women, independent of sexual orientation [e.g., (37)]. To date, only a small number of studies have focused on the behavioral component of body image disturbance in relation to sexual orientation in women. While Wagenbach (29) and Siever (45) reported significantly less investment in one’s own body, such as dieting or exercise (14), in HOW compared to HEW, Cella et al. (30) did not find differences between these groups regarding avoidance behavior. Findings regarding the perceptual component are lacking.

As mentioned above, body image disturbance is a risk factor for the development and maintenance of EDs and is strongly associated with BDD (5, 6, 46). Given this association, the aforementioned findings concerning body image disturbance might reflect disparities in ED and BDD pathology between women with different sexual orientations. Over the course of time, research has focused, among other things, on homosexual orientation as a protective factor for developing eating and weight concerns [e.g., (45, 47, 48)]. However, according to Meneguzzo et al. (49), who investigated the relationship between EDs and sexual orientation in women in a systematic review, none of the examined studies had shown a protective factor against ED symptoms in non-heterosexual women. The authors reported no divergences regarding ED diagnoses in general. However, in terms of ED symptoms, according to the majority of papers as well as a review published by Calzo et al. (50), non-heterosexual women are more likely to show ED symptoms including fasting, dieting, or purging compared to HEW. In contrast, Yean et al. (31) did not find that women differed regarding body image disturbance and ED symptoms depending on their sexual orientation, and Feldman and Meyer (51) reported that lifetime prevalence rates of EDs did not vary in HOW, HEW, and BIW.

Regarding BDD, gender differences in general, and sexual orientation in women in particular, have received little attention in previous research. Boroughs et al. (23) reported more pronounced BDD symptoms in non-heterosexual women than in HEW, and Davids and Green (52) found a higher degree of ED symptoms among bisexual men and women compared to heterosexual and homosexual individuals.

The heterogeneous findings regarding body image disturbance, ED, and BDD symptoms in women with different sexual orientations have been attributed to several factors, including age (53); social context (54), in particular, involvement with the lesbian community (55); and discrimination experience [e.g., Ref. (50)]. Despite changes in body image over the course of an individual’s life span (53, 56), most previous studies examining body image disturbance in women have focused on samples from student populations (57). Moreover, research investigating a possible influence of age on the association between body image disturbance and women’s sexual orientation is mostly lacking. Brown (55) postulated that involvement with the lesbian community might act as a protective factor in the evaluation of one’s own body and the development of a positive body image. Furthermore, the extent of involvement with the lesbian community is negatively correlated with weight concerns (47) and appearance-related concerns (58). By contrast, Beren et al. (59) did not find any relation between involvement with the lesbian community and body image disturbance. Moreover, a report published by the European Union Agency for Fundamental Rights (FRA) showed that individuals with a sexual orientation other than heterosexual still experience high levels of discrimination due to their sexual orientation in different European countries (60), which may endanger the mental health of non-heterosexual individuals [e.g., (61, 62)]. Again, however, studies investigating discrimination as an influencing factor in the relationship between sexual orientation and body image disturbance and associated pathologies are lacking.

In sum, previous studies have shown inconsistent findings regarding body image disturbance and associated pathology of women of different sexual orientations, and BIW have mostly been neglected or integrated into an overall minority group (62). Moreover, past research on this topic has mainly focused on body dissatisfaction, as the cognitive–affective component of body image disturbance. There has been no comprehensive assessment of the broad range of components, and potential influencing factors have largely been disregarded. A deeper understanding of the impact of sexual orientation on body image disturbance would be helpful in order to better tailor existing interventions to individuals and to include previously neglected groups in preventive measures. Therefore, the present study sought to examine the cognitive–affective and behavioral components of body image and associated psychopathology in HOW, HEW, and BIW based on a large data set collected through an online survey. It should be noted that as the survey design did not encompass objective ratings of the participants’ bodies by others, it was not possible to examine the perceptual component of body image. We were also interested in associations of body image components with various potentially relevant factors such as age, experience of everyday discrimination, and involvement with the lesbian community.

Based on the aforementioned findings, we hypothesized that compared to HEW, HOW would show lower scores on drive for thinness, body checking, body avoidance, investment behavior, and body image disturbance as a whole. We further expected that drive for muscularity as well as ED symptoms would be higher in HOW, while HEW would show higher scores on BDD symptoms compared to the other groups. From an exploratory perspective, we investigated differences in drive for leanness. Furthermore, we assumed that compared to HEW, HOW would show a higher number of everyday discrimination experiences, stronger positive associations of discrimination experiences with body image disturbance facets and associated psychopathology, and stronger negative associations of age with these variables. Lastly, we hypothesized that a greater affiliation of HOW with the lesbian community would be negatively correlated with body image disturbance components and psychopathology measures. To complement all of the analyses, we compared the findings from HOW and HEW with the group of BIW from an exploratory perspective.

## Methods

### Recruitment and Participants

Data were collected through an online survey by Unipark (Questback GmbH, Cologne, Germany) including individuals of 18 years or older and with sufficient knowledge of the German language. The sample was recruited from the German-speaking population worldwide from 04/2017 to 09/2018 *via* university e-mail distribution lists, posters and flyers, press releases; lesbian, gay, bisexual, and transgender (LGBT) websites, and Facebook groups. The questionnaire battery used in the current study did not contain any instruments specifically asking for race and ethnicity. However, given the racial and ethnic structure of Germany (63) and the composite of the final sample comprising *n* = 617 female participants, of which the majority had German nationality (*n* = 578), it can be assumed that most of the respondents were Caucasian. Additionally, it is very likely that a large part of the remaining participants were from either Switzerland (64) or Austria (65), countries with similar structures regarding race and ethnicity. The program automatically assesses the IP addresses of the participants. However, this information was not used to prevent repeated participation for the sake of anonymity. Due to the length of the survey of around 40 minutes and no opportunity to skip any questions, however, we assume that no multiple participation occurred. A total of *N* = 6,059 participants of all genders and sexual orientations opened the landing page of the survey, of whom *n* = 1,709 began the survey. The dropout rate was at 30.72%, with *n* = 521 of *n* = 1,709 participants not completing the survey. Out of those who finished the survey, *n* = 424 were excluded due to reporting a sex other than female. Moreover, *n* = 147 women needed to be excluded as they named a sexual orientation other than HOW (*n* = 180), HEW (*n* = 322), or BIW (*n* = 115) (the cell count of other sexual orientations was too low for further analysis).

### Procedure

The study protocol was approved by the university ethics committee. Upon arriving at the landing page of the survey, participants were informed about the aim, duration (around 40 min), privacy, and confidentiality issues of the study; the inclusion criteria; and reimbursement. After they provided informed consent by agreeing to participate, the survey began, and the questionnaires were presented (see below under *Instruments*). After completion, participants were given the opportunity to leave their e-mail address in order to receive a summary of study findings and to enter a lottery to win shopping vouchers.

### Instruments

Below, all instruments reported in the manuscript are listed in alphabetical order. Internal consistencies of all scales employed in the present study were acceptable to excellent (**Table 2**). Additionally, the following instruments were part of the survey but were not included in the present report as they had not yet been validated: Body Image Matrix of Thinness and Muscularity—Female Bodies (Steinfeld et al., in preparation) and Body Parts Evaluation (66; used but not validated in the Cordes study).

*Body Appreciation Scale-2 (BAS-2).* The BAS-2 [Tylka and Wood, (67); revised version of the German-language version of the BAS: (68)] assesses an individual’s general body satisfaction and comprises 10 items.

*Body Image Coping Strategies Inventory (BICSI).* The BICSI [(14); unpublished German translation] identifies how individuals deal with events and circumstances that can threaten their own body image. Only the two subscales *appearance change* (10 items) and *avoidance* (eight items) were used in the present study.

*Body Image Disturbance Questionnaire (BIDQ).* The BIDQ [(69); German-language version, (70)] measures the impact of a negative body image including appearance concern, perceived distress, functional impairment, and avoidance behavior. The questionnaire comprises 12 items, of which seven were included, while the additional five qualitative open-ended items were not used in the present study.

*Contour Drawing Rating Scale* (*CDRS*). The CDRS [(71); German version, (72)] is a silhouette procedure consisting of nine female contour drawings with precisely graduated sizes. Participants are asked to choose the silhouette that most closely resembles the dimensions of their own body. Finally, they are asked to select the silhouette that best represents their own body ideal [cf. Ref. (73)].

*Dysmorphic Concern Questionnaire (DCQ).* The DCQ [(74); German-language version, (75)] is a screening instrument for BDD and comprises seven items.

*Drive for Leanness Scale (DLS).* The DLS [(32), unpublished German translation] is a six-item self-assessment questionnaire to identify the desire for low body fat and visible muscularity (muscle definition).

*Drive for Muscularity Scale (DMS).* The DMS [(33); German-language version] reflects the striving for a more muscular shape. The two subscales *muscle-related cognitions* (seven items) and *muscle-related behavior* (seven items) could not be replicated in women; thus, only the total score is used in the present study.

*Drive for Thinness Scale (DTS).* The seven-item DTS [subscale of the Eating Disorder Inventory [EDI], (76); German-language version, (25)] aims to capture preoccupations with diet and weight and the desire to be thin.

*Eating Disorder Examination-Questionnaire—short version (EDE-Q).* The EDE-Q [German-language version, (77)] (78) is based on the EDE-Interview and captures the psychopathology of EDs. The questionnaire comprises 22 items belonging to the four subscales *eating concern*, *restraint*, *shape concern*, and *weight concern* as well as a global score. The additional six diagnostic items were not used in the present study.

*Gender-Neutral Body Checking Questionnaire (GNBCQ).* The GNBCQ [(79); German version, Waldorf et al., unpublished] assesses body-checking behavior independently of gender.

*Identification and Involvement with the Gay Community Scale—Women’s Version (IGCS-WV).* The eight items of the IGCS-MV [(80); German version, (81), modified from the men’s version] measure the strength of homosexual and bisexual women’s affiliation with the lesbian community.

*Everyday Discrimination Scale (EDS).* The 10 items of the EDS [(82), unpublished German translation] capture the frequency of experience of everyday discrimination.

*Socio-demographic characteristics.* We assessed age, gender, sexual orientation, nationality, relationship status, highest educational attainment, and body height and weight (for the calculation of BMI as kg/m^2^).

### Data Analyses

All analyses were performed using SPSS Statistics Version 24.0 (IBM, Armonk, New York, USA). Differences in demographic, body image, and pathology variables were assessed using Chi-square tests or multivariate analyses of variance with subsequent analyses of variance (ANOVAs) and Bonferroni-corrected *post-hoc tests* with the between-subjects factor group.

To test the postulated group differences in body image components, body image disturbance, ED and BBD pathology, as well as discrimination experience, ANOVAs were conducted. In the case of heterogeneity of variance, Welch’s tests were employed. For ANOVAs, *p*-values were Bonferroni-corrected. Since the three groups differed significantly with regard to age, we additionally conducted analyses of covariance (ANCOVAs) with age as a covariate in order to reduce within-group error variance. Group differences in associations of everyday discrimination experiences, age, and involvement with the lesbian community with body image components and associated ED and BDD pathology were analyzed using Pearson’s correlations. In line with (83), the coefficient *r* can be interpreted as a small effect (*r* = .10), medium effect (*r* = .30), or large effect (*r* = .50). Partial eta-squared was used as a measure of effect size. This indicates the amount of variability explained by the variable that is not explained by any other variable and can be interpreted as a small effect (partial η^2^ ≈.01), medium effect (partial η^2^ ≈.06), or large effect (partial η^2^ ≈.14; 83).

## Results

### Socio-demographic and Anthropometric Characteristics

In total, 617 participants were included in the statistical analyses, of whom *n* = 180 indicated their self-identified sexual orientation as homosexual, *n* = 322 as heterosexual, and *n* = 115 as bisexual. Socio-demographic characteristics of HEW, HOW, and BIW are depicted in **Table 1**. Groups significantly differed in age, with HOW being older than BIW (*p* < .05). Significant group differences occurred regarding relationship status, with *post-hoc tests* illustrating that more HEW (*p* < .001) as well as BIW (*p* < .05) reported being in a relationship than HOW. Groups significantly differed in educational levels, with the group of HEW showing greater percentages of higher attainment compared to HOW in *post hoc* tests (*p* < .005). No group differences between the groups emerged regarding BMI.

**Table 1:** Group comparisons of the three groups regarding demographic characteristics.

Variable		HOW (*n* = 180)	HEW (*n* = 322)	BIW (*n* = 115)	Group compression	*p*
Age: *M*(*SD*)		26.4 (9.21)	24.84 (6.14)	23.98 (6.92)	*F* (2, 263.09) = 3.3	<.05
BMI: *M*(*SD*)		23.95 (9.66)	22.78 (7.19)	23.39 (5.70)	*F* (2, 301.48) = 1.14	.32
Education: *n*					χ² = 15.01	<.05
	University degree/polytechnic degree	48 (26.67%)	118 (36.56%)	39 (33.91%)		
	High school graduation/vocational baccalaureate diploma	108 (60.00%)	187 (58.07%)	63 (54.78%)		
	Secondary school	23 (12.78%)	17 (5.28%)	13 (11.30%)		
	None	1 (0.56%)	–	–		
Relationship: *n*					χ² = 30.23	<.001
	In a relationship^a^	62 (34.44%)	193 (59.94%)	57 (49.57%)		
	Not in a relationship^b^	113 (62.78%)	124 (38.51%)	55 (47.83%)		
	Another unlisted relationship status	5 (2.78%)	5 (1.55%)	3 (2.61%)		

HOW, homosexual women, HEW, heterosexual women, BIW, bisexual women, BMI, body mass index. One-way ANOVAs, by default Welch’s tests (F_w_), with Bonferroni correction as well as Chi-square tests were conducted for the group demographic characteristics. M, mean, SD, standard deviation. ^a^includes committed relationship, married/partnered, living together; ^b^includes single, separated, divorced.

### Group Differences in Body Image Components, Eating Disorder Pathology, and Body Dysmorphic Disorder Pathology

**Table 2** illustrates means, standard deviations, and inferential statistics of group differences. HOW showed significantly lower scores on drive for thinness and drive for leanness than HEW as well as less investment behavior compared to HEW and BIW. Furthermore, both HOW and HEW reported a significantly lower degree of body-checking behavior than did BIW. HOW preferred a significantly larger ideal body size than did HEW. A significant main effect emerged regarding the discrepancy between the actual and ideal figure with respect to body fat, although *post-hoc tests* were not significant. There were no further significant differences in body image components between the three groups. With regard to ED and BDD symptoms, there were no significant differences between HOW, HEW, and BIW. Introducing the covariate age did not change the aforementioned effects, with the exception of the outcome variable body-checking behavior (*F* (2, 613) = 3.731, *p* < 0.05): BIW still reported more body checking compared to HOW (*p* < .05) but no longer differed significantly from HEW (*p* = .773).

**Table 2:** Group comparisons of the three groups regarding body image facets and body image–related pathology.

	Cronbach’s alpha	HOW		HEW		BIW		*F* (df_1_, df_2_)	*p*	**η** ^2^	*post-hoc tests*
		*M* (*SD*)	*n*	*M* (*SD*)	*n*	*M* (*SD*)	*n*				
**Body image facets**											
BAS-2	.94	3.35 (0.88)	180	3.36 (0.78)	322	3.32 (0.82)	115	0.89 (2,614)	.92	.00	Non-sig.
BICSI avoidance	.85	0.94 (0.58)	180	0.86 (0.57)	322	0.98 (0.57)	114	2.05 (2, 613)	.13	.01	Non-sig.
*BICSI appearance change*	.77	1.35 (0.65)	180	1.60 (0.58)	322	1.55 (0.62)	115	9.93 (2, 614)	<.001	.03	HOW < HEW, BIW
CDRS	–	4.44 (1.42)	180	3.95 (1.20)	322	4.27 (1.24)	115	9.11 (2, 614)	<.001	.03	HEW < HOW
DLS	.84	3.32 (1.02)	180	3.63 (0.98)	322	3.39 (1.03)	115	6.20 (2, 614)	<.01	.02	HOW < HEW
DMS	.84	2.13 (0.68)	180	2.14 (0.71)	322	2.20 (0.71)	115	0.32 (2, 614)	.73	.00	Non-sig.
DTS	.93	2.86 (1.38)	180	3.18 (1.27)	322	3.08 (1.35)	115	3.27 (2, 614)	<.05	.01	HOW< HEW
EDS (global score)	.95	2.05 (0.73)	180	1.88 (0.74)	322	2.08 (0.73)	115	4.12 (2, 541)	<.05	.01	Non-sig.
GNBCQ	.76	1.86 (0.55)	180	1.93 (0.03)	322	2.07 (0.58)	115	5.34 (2, 614)	<.01	.02	HEW, HOW < BIW
**Body image–related pathology**											
BIDQ	.90	2.06 (0.94)	173	2.19 (1.91)	312	2.12 (0.84)	104	0.38 (2, 586)	.69	.00	Non-sig.
DCQ	.81	0.93 (0.60)	164	1.01 (0.57)	300	1.07 (0.58)	100	1.86 (2, 561)	.16	.01	Non-sig.
EDE-Q	.76	1.47 (1.33)	162	1.72 (1.30)	297	1.74 (1.28)	98	2.12 (2, 554)	.12	.01	Non-sig.

IGCS-WV	.76	20.41 (4.78)	156	–	–	16.28 (4.52)	98	47.00 (1, 252)	<.001	.16	BIW < HOW

HOW, homosexual women, HEW, heterosexual women, BIW, bisexual women. BAS-2, Body Appreciation Scale-2, BICSI, Body Image Coping Strategies Inventory (two subscales, appearance change and avoidance), CDRS, Contour Drawing Rating Scale, DLS, Drive for Leanness Scale, DMS, Drive for Muscularity Scale, DTS, Drive for Thinness Scale, EDS, Everyday Discrimination Scale, GNBCQ, Gender-Neutral Body Checking Questionnaire, BIDQ, Body Image Disturbance Questionnaire, DCQ, Dysmorphic Concern Questionnaire, EDE-Q, Eating Disorder Examination-Questionnaire, IGCS-WV, Identification and Involvement with the Gay Community Scale—Women’s Version. One-way ANOVAs, by default Welch’s tests (F_w_), with Bonferroni correction were conducted for the group of body image measures and eating disorder pathology separately. M, mean, SD, standard deviation.

### Group Differences in Everyday Discrimination Experience and Involvement With the Lesbian Community

While we found a significant main effect regarding everyday discrimination experience, the *post-hoc tests* did not yield any significant differences between the groups (**Table 2**). HOW reported a significantly greater involvement with the lesbian community than BIW. Again, introducing the covariate age did not significantly change any of the reported findings (all *p* < .05).

### Correlations of Discrimination Experience With Body Image Disturbance, Eating Disorder Pathology, and Body Dysmorphic Pathology

Everyday discrimination experience was positively correlated only with investment behavior in all three groups. In HEW, positive correlations of everyday discrimination experience with body dissatisfaction, drive for muscularity, and body-checking behavior were found. While discrimination experience was positively associated with a higher drive for leanness in HEW and BIW, it was positively associated with avoidance behavior in HEW and HOW (**Table 3**). There was no significant correlation between everyday discrimination experience and drive for leanness or body ideal in any of the groups. Furthermore, the analyses revealed positive associations of discrimination experience with body image disturbance and symptoms of EDs and BDD in all three groups (**Table 3**).

**Table 3:** Pearson’s correlations for everyday discrimination experiences, age, and involvement with the lesbian community in HOW, HEW, and BIW.

	**Discrimination experiences**			**Age**			**Involvement with lesbian community**	
	**HOW** **(** ***n*** ** = 197)**	**HEW** **(** ***n*** ** = 289)**	**BIW** **(** ***n*** ** = 98)**	**HOW** **(** ***n*** ** = 180)**	**HEW** **(** ***n*** ** = 322)**	**BIW** **(** ***n*** ** = 115)**	**HOW** **(** ***n*** ** = 156)**	**BIW** **(** ***n*** ** = 98)**
Body image facets								
BAS-2	−.12	.29**	−.18	.05	.02	.16	.09	−.09
BICSI avoidance	.38**	.22**	.26*	−.08	.07	−.25**	−.06	.08
*BICSI appearance change*	.22**	.38**	.12	−.10	−.08	−.30**	−.00	.02
CDRS	.11	−.01	−.03	.25**	.12*	.11	.18*	.06
DLS	−.08	.00	.01	.06	−.07	−.13	.02	−.13
DMS	.15	.14*	.17	−.07	.03	−.22*	−.02	−.20*
DTS	.03	.31**	.22*	−.04	−.02	−.18	−.12	−.02
GNBCQ	.10	.25**	.17	−.21**	−.22**	−.29**	.10	−.01
Body image–related pathology								
BIDQ	.24**	.16**	.34**	.07	.03	−.22*	−.03	.05
DCQ	.25**	.33**	.26**	−.00	.08	−.26**	−.04	−.05
EDE-Q	.17*	.35**	.29**	−.04	.05	−.05	−.13	−.05

HOW, homosexual women, HEW, heterosexual women, BIW, bisexual women. BAS-2, Body Appreciation Scale-2, BICSI, Body Image Coping Strategies Inventory (two subscales, appearance change and avoidance), CDRS, Contour Drawing Rating Scale, DLS, Drive for Leanness Scale, DMS, Drive for Muscularity Scale, DTS, Drive for Thinness Scale, GNBCQ, Gender-Neutral Body Checking Questionnaire, BIDQ, Body Image Disturbance Questionnaire, DCQ, Dysmorphic Concern Questionnaire, EDE-Q, Eating Disorder Examination-Questionnaire. M, mean, SD, standard deviation.

*p < .05. **p < .01.

### Correlations of Age With Body Image Disturbance, Eating Disorder Pathology, and Body Dysmorphic Pathology

In BIW, age was negatively correlated with investment and avoidance behavior as well as drive for muscularity, while in HEW and HOW, age was positively correlated with a larger ideal body size. Regardless of sexual orientation, age was negatively associated with investment behavior in all three groups. A positive correlation between age and everyday discrimination experience was only found in HEW (**Table 3**). Only in BIW did a significant negative correlation of age with body image disturbance and BDD symptoms emerge. Age was not significantly associated with ED symptoms in any of the groups (**Table 3**).

### Correlations of Involvement With the Lesbian Community With Body Image and Eating Disorder Pathology

With regard to involvement with the lesbian community, the correlation analyses only yielded two significant associations: a positive correlation with a larger ideal body size in HOW and a negative correlation with drive for muscularity in BIW (**Table 3**).

## Discussion

The aim of the current study was to provide a comprehensive assessment of the multidimensional construct of body image disturbance and the associated pathology as well as the influencing effects of age, discrimination experience, and involvement with the lesbian community in HEW, HOW, and BIW. The analyses revealed that HEW reported a greater drive for thinness and leanness and more investment behavior as compared to HOW. However, BIW did not differ significantly from the others in these facets. Furthermore, HOW reported a significantly lower degree of body checking than did BIW, and both HOW and BIW preferred a larger ideal body size compared to HEW. There were no group differences in drive for muscularity, body dissatisfaction, avoidance behavior, and BDD or ED symptoms. With regard to everyday discrimination experience, a significant main effect of sexual orientation was found, although the *post hoc* test did not reveal any specific group differences. In all groups, the greater the experience of everyday discrimination, the more pronounced were the ED and BDD symptoms as well as body image disturbance and investment behavior. Furthermore, discrimination was linked to greater body dissatisfaction, drive for muscularity, and body checking in HEW than in HOW and BIW. For all women, younger age was associated with more body checking. Moreover, while younger age was correlated with more body-related investment and avoidance behavior and a greater drive for muscularity in BIW, older age was associated with a larger ideal body size in HOW and HEW and with more everyday discrimination experiences in HEW. Only in BIW was younger age positively related to BDD pathology as well as body image disturbance. In terms of involvement with the lesbian community, a positive correlation with the ideal body size was found in HOW, and a negative correlation with drive for muscularity emerged in BIW.

Concerning the cognitive–affective body image component, the higher degree of drive for thinness in HEW compared to HOW is in line with most previous research [e.g., (26–29)]. Authors such as Moreno-Domínguez et al. (39) and Swami and Tovée (38) reported significant differences in women’s BMI depending on sexual orientation, which they discussed as a potential reason for the variability in the cognitive–affective body image. As HEW, HOW, and BIW did not differ in BMI in the current study, BMI cannot account for the reported differences in drive for thinness. Both HOW and BIW reported a larger ideal body size compared to HEW. This is consistent with previous research demonstrating that HOW are less influenced by sociocultural standards of beauty, leading to a lower degree of body dissatisfaction and a larger ideal body size (34, 42, 44). According to the present findings as well as previous research, HOW are less concerned about their own weight, leading to a lower drive for thinness, and have a more flexible idea of beauty [e.g., (31, 36, 84, 85)] compared to HEW. In relation to this, drive for leanness, which is presented as the new body ideal (“Strong is the new skinny”) (32), was also lower in HOW compared to HEW in the current study. However, the three groups did not differ in drive for muscularity, which contradicts the findings of Yean et al. (31), who reported a significantly higher drive for muscularity in HOW. In this context, it should be noted that even though the two studies used the same scale to collect data, in the study by Yean et al. (31), HEW were significantly overrepresented in the sample compared to HOW. Moreover, Yean et al. (31) reported that the average BMI differed depending on sexual orientation, with more HOW being overweight or obese than HEW. These differences may have led to the reported higher drive for muscularity in HOW (31).

Concerning the behavioral body image component, HOW showed less investment behavior compared to HEW, which is consistent with the results of Siever (45) as well as Wagenbach (29), who showed that one’s own appearance seems to be less important for HOW than HEW. Even after controlling for age, BIW showed significantly more pronounced body checking than did HOW. In this context, Brewster et al. (86) discussed the impact of antibisexual discrimination and internalized biphobia on the amount of internalization of sociocultural standards of beauty and body surveillance. Since BIW experienced a higher degree of discrimination compared to HEW and HOW, they might have internalized the beauty standards to a greater extent (86). In the current study, BIW did not report more discrimination experiences than HOW and HEW. This discrepancy may be due to the different specificities of the instruments used in the two studies. In contrast to the differences found with respect to body checking, no differences in avoidance behavior were found in the present study. Repetitive checking occurs with the objective of checking that one’s own appearance fully conforms with social and/or personal norms, probably with the aim of decreasing discrimination experiences in the future. Furthermore, we found no differences between BIW and the other two groups regarding drive for thinness, leanness, and muscularity. This is likely due to a mix of genders of the participants’ romantic partners, which has been shown to affect the internalized beauty ideal in BIW (87). A previous study found that BIW with a male partner showed a more traditional feminine body ideal, while BIW with a female partner had a less strictly defined body ideal (88). Accordingly, the gender of the current partner may have led to different body image ideals among the BIW, which in turn may have resulted in the intermediate position of BIW between the two other groups.

In terms of the influence of sexual orientation in women on ED symptoms, two recently published reviews have found that non-heterosexual women have greater ED symptoms than HEW (49, 50), while others reported no significant differences [e.g., (30, 50)]. In the current study, women did not differ in pathological symptoms regarding sexual orientation. We concluded, in line with, for example, Share and Mintz (85) as well as Feldman and Meyer (51), that the general societal preference of a thin body and concomitant high body image standards, and therefore the risk for EDs, are equal in women regardless of their sexual orientation. However, since the sample of the study is mostly German, these results may only apply to patterns of ED symptoms in Western cultures. The impact of acculturation-related variables on ED pathology and sexual orientation are interesting topics for further studies since research has already underscored the significance of cultural influence on body image dissatisfaction and developing ED symptoms [e.g., (23, 89)].

In terms of everyday discrimination experience, a positive association with body image–related pathology was found regardless of women’s sexual orientation. In contrast to findings from the European Union Agency for Fundamental Rights (60), in the current study, BIW did not report more discrimination experiences than the other women. We found that the greater the experience of discrimination, the more pronounced was the investment behavior in all three groups. Additionally, a greater experience of discrimination was linked to stronger effects on body image facets in HEW compared to BIW or HOW. It is possible that HOW and BIW are more used to discrimination than HEW in general, and that they attribute these experiences to internal, stable characteristics of their sexual orientation rather than to their appearance. This, combined with their lower degree of internalization of stereotypically feminine beauty ideals evoked by the media (90), may provide an explanation for this lack of association.

Regarding the association between age and body image components, we found that younger age was positively associated with more body checking irrespective of sexual orientation. In comparison to BIW, with increasing age, HOW and HEW showed a greater preference for a body ideal with significantly more body fat. This is in line with the findings of Tiggemann (53), who reported that the relevance of figure, weight, and appearance decreases over time.

In terms of involvement with the lesbian community, a positive association with a larger ideal body size was only found in HOW. This is in accordance with previous studies reporting that involvement with the lesbian community is related to fewer weight concerns (47), fewer appearance concerns (58), and more acceptance of different body shapes (91) in HOW. Such an association is lacking in BIW, possibly due to the impact of biphobia, a specific form of discrimination, stereotypes, and stigma (92) held by both HEW and HOW towards BIW (93), which may lead to BIW feeling less protected by involvement with the lesbian community. Moreover, greater involvement with the lesbian community was associated with a lower drive for muscularity in BIW. It may be that BIW try to dissociate themselves from the “masculine stereotype” held about HOW, with a lower drive for muscularity leading them to feel a greater belonging to the lesbian community (94). A lower drive for muscularity relates to a feminine body ideal, with which most BIW identify themselves [e.g., Ref. (95)]. For all of the other body image components and related pathology, no associations with involvement in the lesbian community were found in HOW and BIW.

## Limitations and Conclusion

The results of the present study need to be interpreted in light of some limitations pertaining to sample and design. The three groups differed in size, although this is in line with the different distributions of HEW, HOW, and BIW in the general population. Additionally, there were differences regarding age and educational level, which may explain some of the variance in the body image components. Nevertheless, statistically controlling for these differences did not significantly change the results. Since only women with a minimum age of 18 years were eligible to participate in the present study, the findings cannot be generalized to female adolescents. Future studies should therefore include female adolescents in order to capture the crucial point of coming out during a phase that is already relevant for the development of body image (96). Moreover, the study only included individuals with sufficient German language skills, which may limit the generalizability of the findings to different nationalities and cultures. Additionally, the results of the current study were compared to evidence collected across different cultures and countries, wherefore aforementioned distinctions between these study results may be attributed to the varying cultural settings each study was undertaken in. Among other reasons for why body image disturbance in general and EDs in particular could underlie a cultural impact are the suggestion that non-Western societies traditionally do not value a thin body ideal (97, 98) and that a collectivistic instead of an individualistic structure of society provides a certain degree of protection for its members (98). Furthermore, we only examined a non-clinical, mostly academic sample, thus limiting the ability to generalize the findings to a non-community-based population.

Although online surveys entail many advantages, such as time and cost efficiency or independence of location (99), they are also subject to some weaknesses, such as the inclusion only of participants who have a computer and Internet access. However, it is possible that only within this safe and anonymous context did participants feel able to answer sensitive questions regarding sexual orientation and body image openly and honestly (100). As the present study used a quasi-experimental design, it was only possible to report associations between sexual orientation and body image disturbance as well as related pathology in women. Furthermore, only explicit measurements like self-report questionnaires but not interviews or experimental paradigms, e.g., making use of eye-tracking technology, were used in the current study. This may have led to participants selectively suppressing information. Finally, we did not assess the perceptual component of body image disturbance, as due to the study design, we did not have objective ratings of the participants’ bodies with which to compare individual, subjective ratings.

Despite these limitations, the present study is the first to comprehensively investigate body image disturbance, associated psychopathology, and potential influencing factors in women with different sexual orientations. In particular, due to the inclusion of a large sample of BIW, a subgroup that has often been neglected in previous research, the study contributes differentiated insights into the aforementioned issues. The main differences in HEW and HOW emerged in the cognitive–affective component of body image, with lower pathology in HOW. Regarding the behavioral component, we found a higher degree of body checking in BIW. In conclusion, although we did not find an increased vulnerability to a negative body image based on sexual orientation, differences did emerge between the three groups regarding facets of body image disturbance, suggesting that single facets or aspects of body image might hold differential relevance for the different groups, and that social context and discrimination experience may influence body image. Additionally, since body image disturbance, ED symptoms, and BDD symptoms are known to have a crucial impact on women across age, ethnicities, cultures, and socioeconomic levels (101), it seems to be important to take sexual orientation into account in order to understand the development of body image and body image disturbance in detail and to create optimally suitable prevention measures for women.

## Data Availability

The datasets generated for this study are available on request to the corresponding author.

## Ethics Statement

This study was reviewed and approved by Osnabrück University Ethics Committee (Ethikkommission der Universität Osnabrück). The patients/participants provided their written informed consent to participate in this study.

## Author Contributions

ATH analyzed the data and wrote the first draft of the manuscript. CT, ASH, and SV planned the study, and critically edited the manuscript. CT conducted the recruitment and the study.

## Funding

The authors declare that they obtained no funding for the conduct of the study. We acknowledge support by Deutsche Forschungsgemeinschaft (DFG) and Open Access Publishing Fund of Osnabrück University for the publication of the article.

## Conflict of Interest Statement

The authors declare that the research was conducted in the absence of any commercial or financial relationships that could be construed as a potential conflict of interest.
